# DPP-4 Inhibitors in the Prevention/Treatment of Pulmonary Fibrosis, Heart and Kidney Injury Caused by COVID-19—A Therapeutic Approach of Choice in Type 2 Diabetic Patients?

**DOI:** 10.3389/fphar.2020.01185

**Published:** 2020-08-05

**Authors:** Andrija Smelcerovic, Gordana Kocic, Mihajlo Gajic, Katarina Tomovic, Vukica Djordjevic, Dobrila Stankovic-Djordjevic, Marko Anderluh

**Affiliations:** ^1^ Department of Chemistry, Faculty of Medicine, University of Nis, Nis, Serbia; ^2^ Department of Biochemistry, Faculty of Medicine, University of Nis, Nis, Serbia; ^3^ Department of Pharmacy, Faculty of Medicine, University of Nis, Nis, Serbia; ^4^ Department of Microbiology and Immunology, Faculty of Medicine, University of Nis, Nis, Serbia; ^5^ Department of Pharmaceutical Chemistry, Faculty of Pharmacy, University of Ljubljana, Ljubljana, Slovenia

**Keywords:** COVID-19, diabetes, DPP-4 inhibitors, fibrosis, multi-organ injury

## Abstract

Since the outbreak of SARS-CoV-2 virus more than 12,500,000 cases have been reported worldwide. Patients suffering from diabetes and other comorbidities are particularly susceptible to severe forms of the COVID-19, which might result in chronic complications following recovery. Dipeptidyl peptidase-4 inhibitors exert beneficial effects in prevention/treatment of pulmonary fibrosis, heart, and kidney injury, and since they may be a long-term consequence caused by COVID-19, it is reasonable to expect that DPP-4 inhibitors might be beneficial in alleviating long-term consequences of COVID-19. With that in mind, we would like to voice our concerns over chronic implications following recovery from COVID-19, especially not only in diabetic but also in non-diabetic patients, and to indicate that some preventive measures could be undertaken by application of DPP-4 inhibitors.

## Introduction

The end of SARS-CoV-2 pandemic is still behind the horizon, as the total confirmed cases number rapidly rises. As of July 12, 2020, more than 12,500,000 cases of SARS-CoV-2 infections have been reported, out of which more than 560,000 ended lethally (World Health Organization, 2020). The presence of chronic comorbidities may aggravate clinical findings and induce fatal outcome, as evidenced by the study on 44,672 confirmed cases, out of which 1023 had fatal outcome. Case fatality rates reported for patients suffering from chronic comorbidities including cardiovascular disease, diabetes, chronic respiratory disease, and hypertension (10.5%, 7.3%, 6.3,% and 6.0%, respectively) were much higher compared to case fatality a rate of 0.9% for patients without comorbid conditions (Novel Coronavirus Pneumonia Emergency Response Epidemiology Team, 2020). With the main focus of public health authorities on transmission prevention and development of vaccines and treatments, long-term consequences on the health of recovering and recovered patients remain subordinate. Radiological findings of COVID-19 patients indicate to pneumonia associated with rapidly developed pulmonary fibrotic changes, even in asymptomatic patients (Shi et al., 2020). Based on similarities between ongoing and previous coronavirus infections, excessive lung damage accompanied by fibrosis might result in functional disabilities, decreasing quality of life among survivors (Ngai et al., 2010; Batawi et al., 2019). The evolution of pneumonia to pulmonary fibrosis may vary, but the interval between the onset of symptoms and the development of pulmonary fibrosis may be relatively short, sometimes even just a few days (Shi et al., 2020), which indicate an urgent therapeutic approach to preserve pulmonary function. Besides, multi-organ injury affecting liver, kidneys, and heart, among others, which may eventually result in organ failure, is quite common among COVID-19 patients in intensive care units (Wang et al., 2020). SARS-CoV-2 either induces new cardiac pathologies, such as myocarditis, and/or leads to the exacerbation of existing ones (Madjid et al., 2020), implying potential long-term cardiovascular effects of COVID-19. This might be important, especially for diabetic patients who already suffer from reduced lungs, hearth, and kidney function, making them particularly susceptible to cumulative organ injury during infection with SARS-CoV-2. With that in mind, we would like to voice our concerns over chronic implications following recovery from COVID-19, especially not only in diabetic but also in non-diabetic patients, and to indicate that some preventive measures could be undertaken by application of DPP-4 inhibitors.

## Implications of DPP-4 Inhibition in Multi-Organ Injury Caused by COVID-19

The potential role of DPP-4 in the pathogenesis of COVID-19 is a subject of the ongoing debate. Various authors emphasized anti-inflammatory properties of DPP-4 inhibitors with conflicting opinions on whether these effects are beneficial or detrimental in patients with COVID-19 (Bouhanick et al., 2020; Chen C. F. et al., 2020; Dalan, 2020; Drucker, 2020; Iacobellis, 2020; Kokic Males, 2020; Pinheiro et al., 2020). In addition, Strollo and Pozzilli (2020) pointed to well-known anti-fibrotic activity, while Bassendine et al. (2020) further expanded on the topic by reflecting on cardioprotective effects of DPP-4 inhibitors. However, considerations made in both articles regarding the involvement of DPP-4 as a viral entry receptor, which are based on *in silico* predictions (Vankadari and Wilde, 2020), are highly unlikely and in contradiction with experimental evidence (Letko et al., 2020; Zhou et al., 2020).

Amplified and aberrant immune response to SARS-CoV-2 infection causes pulmonary injury triggering defensive pro-fibrotic mechanisms, as a result of simultaneous tissue remodeling by activated myofibroblasts and migrating fibroblasts, which possess on their surface dipeptidyl peptidase-4 (DPP-4)/CD26. In fact, inflammation is tightly interconnected with fibroblast activation, migration, and proliferation. However, although host immune response has crucial role in COVID-19 pathology, opinions on the use of anti-inflammatory therapies, especially in critically ill patients, are conflicting (Ritchie and Singanayagam, 2020).

**Figure 1:**
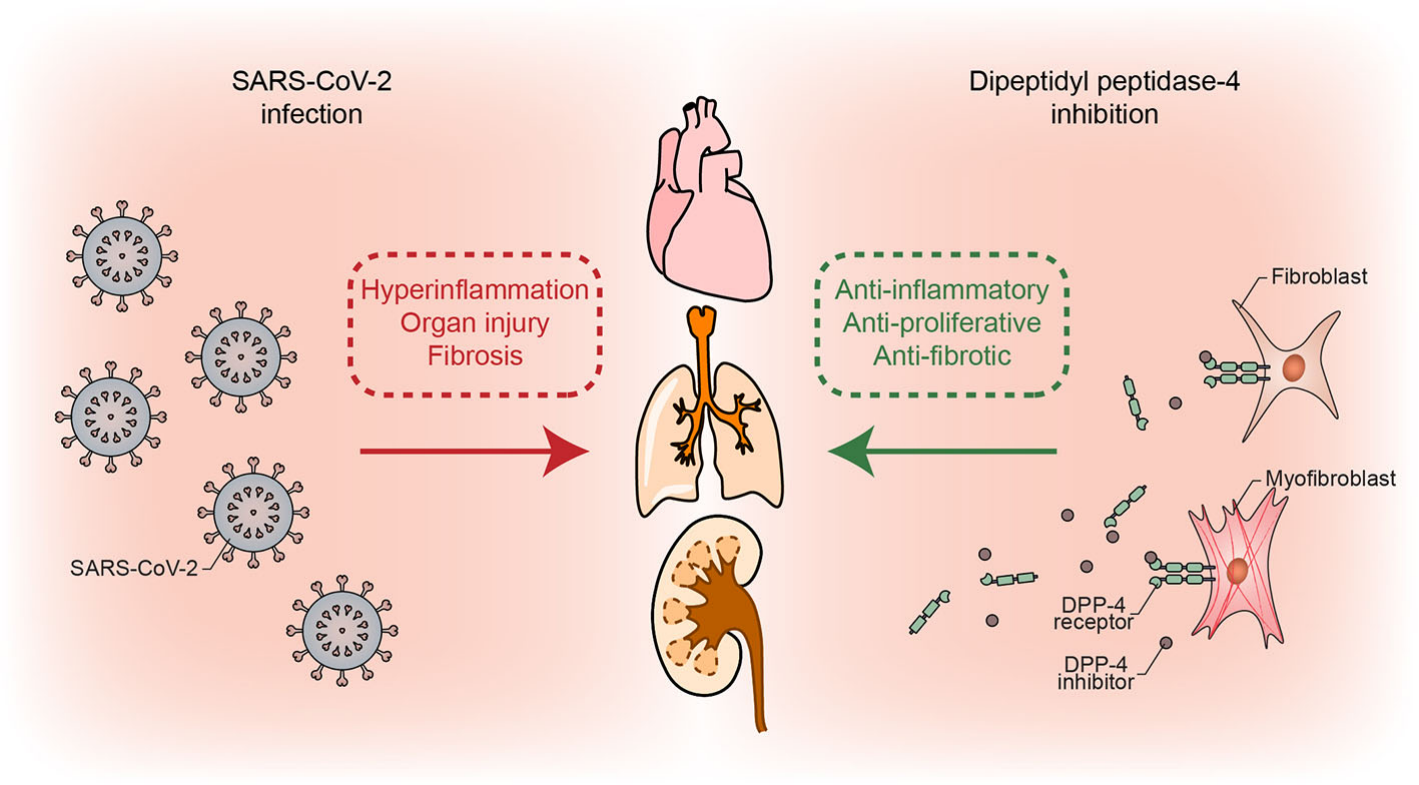
Simplified mechanism of possible beneficial effects of DPP-4 inhibition through its anti-inflammatory, anti-proliferative, and anti-fibrotic actions, which counteract detrimental COVID-19 repercussions.

DPP-4 inhibitors are able to suppress inflammatory signalling and proliferation of vascular smooth muscle cells (Xu et al., 2018), which are important players in the reversible phase of pulmonary vascular remodeling. We have indicated the possibility that DPP-4 inhibitors might prevent fibrosis and delay or suppress the entry to the irreversible phase of vascular remodeling in pulmonary hypertension by reducing the activity of pro-fibrotic mediators, proliferation, and migration of fibroblasts (Anderluh et al., 2019). These pleiotropic anti-inflammatory effects of DPP-4 inhibitors are incomparable to standard anti-inflammatory therapies (i.e., corticosteroids) and might be useful in the therapy of COVID-19 due to their other favorable effects, especially after the clinical findings, suggesting the lack of benefit from corticosteroids and even indicating to deleterious effects accompanied with late complications (Russel et al., 2020). **Figure 1** depicts the proposed mechanism of beneficial effects of DPP-4 inhibition on SARS-CoV-2 damaged lungs and other organs.

In addition, we have pointed to many beneficial pleiotropic effects of DPP-4 inhibitors with protective role in renal and cardiovascular disorders as specific entities or type 2 diabetes mellitus associated comorbidities including myocardial regeneration. DPP-4 inhibitors exert these effects by reducing degradation of stromal cell–derived factor-1, which enhances homing of endothelial progenitor cells and ameliorates functional myocardial reparation outcome (Anderluh et al., 2016; Tomovic et al., 2019).

Recent reports clearly indicate that both hypertension and diabetes represent special risk factor as comorbidities with COVID-19 (Novel Coronavirus Pneumonia Emergency Response Epidemiology Team, 2020). Accordingly, Fang et al. (2020) continued to suggest that patients with hypertension, who are prescribed either ACE inhibitors or angiotensin receptor antagonists known to increase ACE2 expression, might be at greater risk for severe COVID-19 infection and, therefore, should be monitored for ACE2-modulating medications. In contrast with ACE2-modulating medications, we have offered the hypothesis that DPP-4 inhibitors act in prevention/treatment of pulmonary fibrosis, heart, and kidney injury, and since they may be a long-term consequence caused by COVID-19, it is reasonable to expect that DPP-4 inhibitors might be beneficial in alleviating long-term consequences of COVID-19. Fadini et al. (2020) conducted a case-control study revealing that T2DM patients with COVID-19 had similar disease outcomes, regardless of therapy with DPP-4 inhibitors. Nonetheless, they advocate to DPP-4 inhibition as a valid therapeutic option due to optimal safety profile in comparison to other T2DM medications. Since DPP-4 inhibitors are one of the mainstream medications for the treatment of diabetes mellitus type 2, with demonstrated safety and tolerability (Deacon, 2019; Chen Y. et al., 2020; Fadini et al., 2020), it would be rather undemanding to monitor the effect of DPP-4 inhibitors on long-term consequences on the health of COVID-19 recovering or recovered patients. We urge the scientific community to do exactly this.

## Conclusion

In conclusion, the main purpose of this article is to raise the awareness that the end of SARS-CoV-2 pandemic will not necessarily be the end of our fight with COVID-19, since the long-term consequences of excessive lung, heart, and kidney injury in continuously increasing number of patients are yet to be seen. To the best of our knowledge, we are the first to hypothesize that DPP-4 inhibitors might emerge as a valuable asset in the alleviation of COVID-19 long-term repercussions following patients’ recovery, given their favorable effects in protection and tissue regeneration. Administration of DPP-4 inhibitors to COVID-19 patients with ongoing type 2 diabetes mellitus could be advisable, as they are capable to benefit from effects on glycemic control, but also from protective anti-inflammatory, anti-proliferative and anti-fibrotic effects in the lungs, hearth, and kidneys. At the end of twentieth century the concept of ʹʹone molecule-one target-one diseaseʹʹ shifted toward multitarget drugs able to control complex diseases (Alcaro et al., 2019). DPP-4 inhibitors are capable of simultaneously treating both diabetes and its multiple complications. The mentioned effects of DPP-4 inhibitors are expected to be beneficial for some or even all SARS-CoV-2–infected patients, and we propose them as potential adjunctive treatment option aimed to alleviate COVID-19 long-term complications, especially knowing that millions of people will likely be affected by COVID-19.

## Author Contributions

AS, GK, and MA designed manuscript. MG, KT, DS-D, and VD conducted literature search. All authors contributed to the article and approved the submitted version.

## Funding

The financial support of this work by Ministry of Education, Science and Technological Development of the Republic of Serbia; Serbian Academy of Sciences and Arts, branch in Niš (project О-06-17); Faculty of Medicine of the University of Niš (internal project no. 40); and Slovenian Research Agency (grant P1-0208) is gratefully acknowledged.

## Conflict of Interest

The authors declare that the research was conducted in the absence of any commercial or financial relationships that could be construed as a potential conflict of interest.
